# Loneliness and cognitive function in later life: a population-based counterfactual analysis

**DOI:** 10.1093/ije/dyag212

**Published:** 2026-10-06

**Authors:** Shubhankar Sharma, Jo Mhairi Hale, Angelo Lorenti, Solveig Cunningham

**Affiliations:** Faculty of Social Sciences, University of Helsinki, Helsinki, Finland; Helsinki Institute for Demography and Population Health, Max Planck–University of Helsinki Centre for Social Inequalities in Population Health, Rostock, Helsinki, Germany, Finland; School of Geography and Sustainable Development, University of St Andrews, St Andrews, United Kingdom; Helsinki Institute for Demography and Population Health, Max Planck–University of Helsinki Centre for Social Inequalities in Population Health, Rostock, Helsinki, Germany, Finland; Labor Demography, Max Planck Institute for Demographic Research, Rostock, Germany; Health, Ageing and Mortality, Netherlands Interdisciplinary Demographic Institute, Hague, Netherlands

**Keywords:** loneliness, depression, cognitive function, gender, life course, parametric g-formula, causal inference, mediation

## Abstract

**Background:**

Loneliness is increasingly recognized as a major public health challenge. Yet, its impact on cognitive function remains poorly understood. A complex interplay of life-course risk factors makes it challenging to disentangle the effect of loneliness from other factors and to assess underlying mechanisms when using traditional approaches.

**Methods:**

We applied the parametric g-formula with the Health and Retirement Study (1996–2018; *n* = 34 774; aged 50–80 years) to simulate an intervention estimating the effect of preventing loneliness on cognitive function. We assessed whether this effect varies by gender. Formal mediation analysis was also performed to assess depression as a potential pathway.

**Results:**

Preventing loneliness was protective against cognitive decline at all ages, with the magnitude increasing with age. Averaged across all ages, an intervention to prevent loneliness would slow cognitive decline by 0.075 points at the population level [95% confidence interval (CI): 0.063–0.089] and by 0.157 points among individuals experiencing loneliness (95% CI: 0.138–0.178). Overall, 17.3% of the total effect of preventing loneliness was mediated via depression, with the proportion mediated being 19.4% among men and 16.9% among women.

**Conclusion:**

Employing an innovative counterfactual framework, this study shows that preventing loneliness could provide an insulative effect against cognitive decline, even at the population level. Importantly, with advancing age, addressing loneliness yields progressively greater cognitive benefits. The effects partly operate through reductions in depression, especially in men. Interventions against loneliness may help maintain cognitive function, with benefits extending to mental health, highlighting a promising avenue for public health strategies.

Key Messages

- Due to complex interdependencies between life-course processes, it remains unclear how loneliness and depression affect later-life cognitive decline.
- Using an innovative counterfactual approach, we find that preventing loneliness protects against cognitive decline and that depression acts as an important mediating pathway, particularly for men.
- Loneliness and depression can be understood as modifiable risk factors for later-life cognitive function and so reducing loneliness offers a promising strategy to jointly preserve cognitive and mental health.

## Background

Loneliness has emerged as one of the greatest public health challenges in modern societies [1], carrying a mortality risk that is comparable to those for smoking, obesity, and diabetes [2]. Loneliness is the subjective unpleasant feeling resulting from a gap between desired and actual social relationships [3]. It differs from social isolation, which is an objective measure of minimal social contacts and interactions, with both independently related to health [4, 5]. Meanwhile, the global cognitive impairment burden is growing with population aging; 57 million people are estimated to be living with dementia (severe cognitive impairment)—a number expected to nearly triple by 2050 [6]. Delaying dementia onset and progression by just 1 year could reduce global cases by >9 million by 2050 [7], highlighting the urgent need to identify modifiable risk factors.

Later-life cognitive function is shaped by social, educational, employment, partnership, and health-related factors across the life course [8]. Most of these predictors of cognitive function are also associated with loneliness [9]. Nevertheless, the relationship between loneliness and cognitive function remains poorly understood. Some studies report an increased risk of cognitive impairment and faster cognitive decline among lonely individuals, while others find no significant association [5, 10]. Evidence on whether this relationship differs by gender also remains unclear [4, 11]. Much of the evidence relies on cross-sectional observations [10]. The inconsistencies may reflect heterogeneity in measurements, samples, and analytical approaches.

A variety of direct and indirect mechanisms may underlie the relationship between loneliness and cognitive functioning. Loneliness diminishes executive function by causing repeated activation of implicit hypervigilance for social threat [12]. Biologically, loneliness is associated with heightened activation of the hypothalamic–pituitary–adrenal axis, increased inflammation, and reductions in dendritic arborization, prefrontal–cortico–striatal functioning, and brain-derived neurotrophic factors, all of which may negatively impact cognitive function [12]. Indirectly, loneliness may also lead to reduced engagement in cognitively stimulating activities, such as visiting museums and playing games [13], which may diminish neural reserves, increasing vulnerability to neuropathology. Depression may act as a pathway: lonely individuals are more likely to develop depression—an association stronger among men [14]. Depression, in turn, is linked to poorer cognitive function [8].

A key challenge in understanding this relationship is the complex interplay of life-course processes over time. The pathways to cognitive function and loneliness are intertwined and can form reciprocal relationships, complicating effect estimation when using traditional regression methods [15]. Standard regression-based adjustment also provides biased estimates when time-varying confounders are affected by prior exposure [16, 17]. Depression, which may link loneliness and poor cognition, can itself increase the risk of both, creating intermediate confounding, complicating assessment of the mediating role of depression when using standard approaches [18]. Moreover, because cognitive impairment may lead to depression [8], controlling for depression could introduce collider bias [18], spuriously linking loneliness to cognitive function.

The parametric g-formula provides a powerful framework to overcome these complexities. It enables modeling of time-varying risk factors, allowing longitudinal interdependencies, intermediate confounding, mediation, reverse causality, and selection [16, 19–22]. A key advantage of this approach is its ability to evaluate how population-wide policy actions, such as preventing loneliness, may affect the wider population. The scarcity of evidence on how loneliness interventions might influence cognitive function is a critical literature gap [5]. Primary prevention of loneliness may be particularly important, as the cognitive harms of becoming lonely outweigh the benefits of recovery [23]. Modeling a scenario in which loneliness is avoided provides a benchmark for the maximum potential cognitive benefits at the population level.

Using this advanced counterfactual framework and the high-quality longitudinal Health and Retirement Study (HRS), we address three research questions: (i) Does preventing loneliness affect cognitive function? (ii) Does this effect vary by gender? (iii) To what extent is the effect mediated via depression? These novel insights can support preventive social strategies for a medical condition that currently has no cure.

## Methods

### Data

We use data from the HRS, which is a nationally representative longitudinal survey of US adults aged >50 years and their spouses of any age. Interviews were conducted biennially, with baseline interviews primarily conducted face to face and follow-up interviews conducted via telephone or face to face. Proxy interviews were used when respondents were unable or unwilling to participate. Analyses are based on the RAND-HRS Longitudinal File 2018 (V2), covering 12 waves (1996–2018) [24]. We include respondents with data on loneliness, depression, and cognitive function during 1996–2018 and with non-missing information on covariates (all covariates have <5% missing). We exclude adults aged >80 years due to smaller sample sizes in this age group, which leads to irregularities in cognitive function patterns and reduces estimation accuracy. Our final analytical sample comprises 34 774 adults aged 50–80 years, contributing 179 886 person-waves.

### Measures

#### Cognitive function

Cognitive function is assessed by using a modified version of the Telephone Interview for Cognitive Status test. We include tasks reflecting “fluid” cognitive abilities, summing immediate (0–10 points) and delayed (0–10 points) word recall, serial sevens (0–5 points), and counting backward (0–2 points), for a range of 0–27; higher scores indicate better cognition.

#### Loneliness

Loneliness is treated as a binary variable (yes/no), embedded in the Center for Epidemiological Studies-Depression (CES-D) scale, asking whether respondents felt lonely much of the time during the past week. This single-item measure is widely used as a direct measure of loneliness and correlates strongly with multi-item loneliness scales [25]. It has a lower response burden, making it more appropriate in studies involving older individuals [26]. We offer a detailed discussion on the selection of this measure in the Supplementary Materials.

#### Time-varying covariates

Time-varying covariates included age; partnership status [partnered (partnered/married) vs unpartnered (separated/divorced/spouse absent/widowed/never married)]; employment status [employed (full-/part-time work), retired (retired/partly retired), or inactive (unemployed/disabled/not in labor force)]; depressive symptoms (none or one vs at least two CES-D symptoms, excluding “loneliness”) [4]; and comorbidities (none vs one or more self-reported diagnoses of diabetes, stroke, hypertension, and/or heart condition).

#### Time-constant covariates

Time-constant characteristics are gender (1 = women), race/ethnicity (non-Latinx White, Latinx/non-Latinx Black, non-Black Latinx, and non-Latinx Others), and educational attainment (less than high school, high school/general equivalency degree/some college, and college and higher).

### Statistical analysis

We use the parametric g-formula, which is a simulation-based counterfactual approach, in which “simulation” refers to generating longitudinal trajectories of time-varying variables for each individual by using the estimated regression models under both the observed (natural-course) and hypothetical (e.g. preventing loneliness) intervention scenarios.

This simulation-based framework, unlike traditional regression approaches, enables us to assess the impact of preventing loneliness on cognitive function, accounting for how preventing loneliness would impact all the time-varying characteristics influenced by loneliness itself. In this way, this simulation framework captures how changes in the exposure (loneliness) propagate through related processes over time, producing cascading effects across time-varying characteristics [22]. This enables a more realistic assessment of the role of the interventions in the changes in the outcome (cognitive function). As with other models, the g-formula has a set of underlying assumptions. Full discussion of those assumptions can be found in the Supplementary Materials.

The parametric g-formula is implemented in four steps. First, based on literature, we draw a causal directed acyclic graph (DAG) describing hypothesized relationships between the variables (see Supplementary Figures 1 and 2). The Supplementary Materials also include a discussion of the literature informing the DAG. We use a cross-lagged design in which variables at age t influence those at age t+2, establishing temporal order and reducing biases due to reverse causation. The assumed time lag between loneliness and cognitive function aligns with existing literature using biennially spaced data, demonstrating that loneliness predicts lower cognitive performance in the next wave [27, 28]. All time-varying variables are treated as mediators, confounders, and outcomes. Second, following the DAG, we estimate multivariable models for the exposure, outcome, and time-varying confounders. Third, we define a hypothetical intervention scenario: what if lonely adults were not lonely? Fourth, we start with the observed data at the first time point and the estimated multivariable models to simulate all time-varying variable values at subsequent follow-up times. Repeating this process across the follow-up times allows us to reproduce the population’s observed longitudinal trajectory, known as the “natural-course scenario.” We implement an “intervention scenario,” which is similar to the natural-course scenario with the exception that, in the simulation, all individuals who are lonely in the natural-course scenario are instead set to non-lonely throughout the follow-up. The difference in average cognitive function scores between these scenarios represents the total effect (TE) of preventing loneliness [19]. We also estimate the TE by gender.

We interpret the TE at the population level (population average effect), reflecting the protective effect of changing the 16% of lonely person-waves to non-lonely, while the remaining 84% (already non-lonely) still contribute to the denominator, diluting the overall estimate. We therefore also estimate the average treatment effect on the treated (ATT), which focuses only on those ever experiencing loneliness, capturing the effect of a targeted intervention.

Finally, following Wang and Arah (2015) [19], we estimate the indirect effect (IE) of preventing loneliness operating through depression and the direct effect (DE) operating via other mechanisms. We estimate confidence intervals (CIs) by using non-parametric bootstrapping with 500 resamples, maintaining the longitudinal structure of the data [21, 22]. Each bootstrap iteration includes 50 simulations, to minimize variation due to the Monte Carlo simulations. We provide a detailed description of the g-formula implementation in the Supplementary Materials.

## Results

Table 1 presents descriptive statistics of the analytical sample. Of 179 886 person-waves, 16.2% report loneliness, with women (18.6%) more likely to than men (12.8%). Lonely adults have lower average cognitive scores (13.9 vs 15.9), with similar mean ages across the groups. Adults with two or more depressive symptoms are about seven times more likely to feel lonely than those with at most one. Loneliness is about twice as prevalent among adults who are Black, Latinx, and have less than high-school education than among adults who are White and have an education of college or above. Inactive adults are about two times more likely to experience loneliness than those who are employed. Unpartnered adults are three times more likely to feel lonely than partnered adults.

**Table 1:** Descriptive characteristics of the sample over the study period 1996–2018.

	No Loneliness	Loneliness	Person-waves
Mean cognitive score	15.9	13.9	179 886
Age (years) (average)	64.6	64.7	
Sex			
Women	81.4	18.6	105 423
Men	87.2	12.8	74 463
Race/ethnicity			
White	86.5	13.5	123 255
Black	79.1	20.9	31 572
Latinx	75.3	24.7	19 832
Other	81.3	18.7	5227
Education			
Less than high school	73.8	26.2	36 042
High school/general equivalency degree/some college	84.5	15.5	95 325
College+	89.9	10.1	48 519
Depressive symptoms			
0 or 1	94.5	5.5	124 579
≥2	59.8	40.2	55 307
Labor-force status			
Employed^a^	88.7	11.3	61 096
Retired^b^	83.1	16.9	96 656
Inactive^c^	73.4	26.6	22 134
Partnership status			
Partnered	90.2	9.8	121 538
Unpartnered	70.6	29.4	58 348
Comorbidity			
None	87.3	12.7	66 558
One or more	81.8	18.2	113 328
Total	150 768	29 118	179 886

a Full-time/part-time work.

b Retired/partly retired.

c Unemployed, disabled, or not in the labor force.

Supplementary Figure 3 shows that the age trajectories of cognitive function in the natural-course and empirical scenarios align closely. Most importantly, the natural-course scenario appropriately reflects the observed cognitive decline by age. The average cognitive function decreases from ∼16.5 to 13 points between ages 50 and 80 years.

### TE of preventing loneliness at the population level

The age trajectories of average cognitive function under the intervention and natural-course scenarios are compared in the upper panel of Fig. 1. The lower panel shows the age trajectory of the difference between scenarios—the TE at the population level.

**Figure 1:**
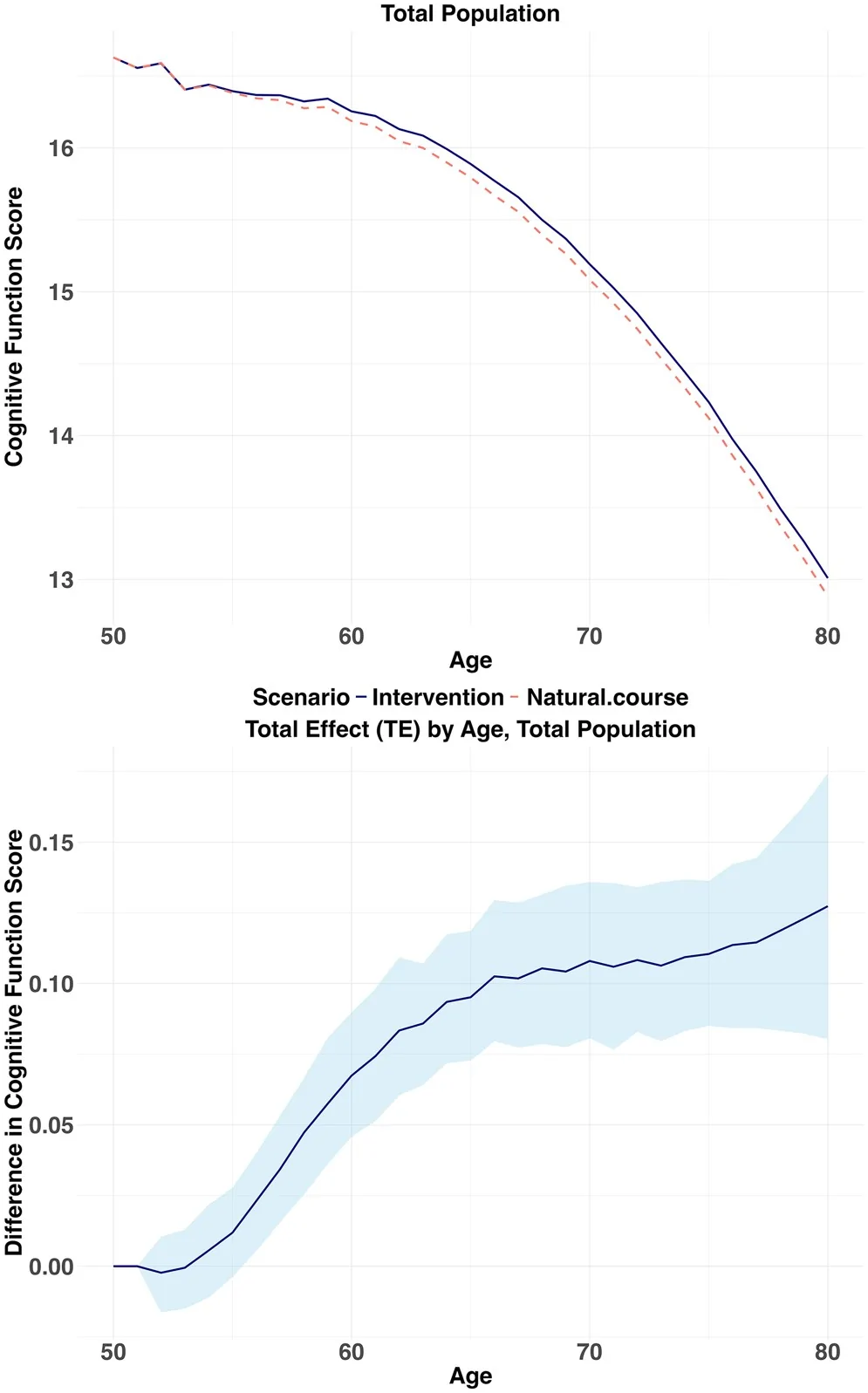
Age-specific comparison of the natural-course and intervention scenarios (top panel) and their difference at the population level, with 95% CIs, for total population aged 50–80 years in the USA, 1996–2018 (bottom panel).

Our findings suggest that preventing loneliness could protect against cognitive decline, with this insulative effect increasing with age. Averaged across all ages, the intervention slows cognitive decline by 0.075 points (95% CI: 0.063–0.089) at the population level.

### ATT

Figure 2 shows the effect of preventing loneliness only among those who experience loneliness, i.e. the ATT, which is expected to be greater than the TE, as it isolates the effect on the affected subgroup. The ATT shows an increasing trend with age, before declining slightly after age 70 years. When averaged across all ages, the intervention slows cognitive decline by 0.157 points (95% CI: 0.138, 0.178) among those experiencing loneliness.

**Figure 2:**
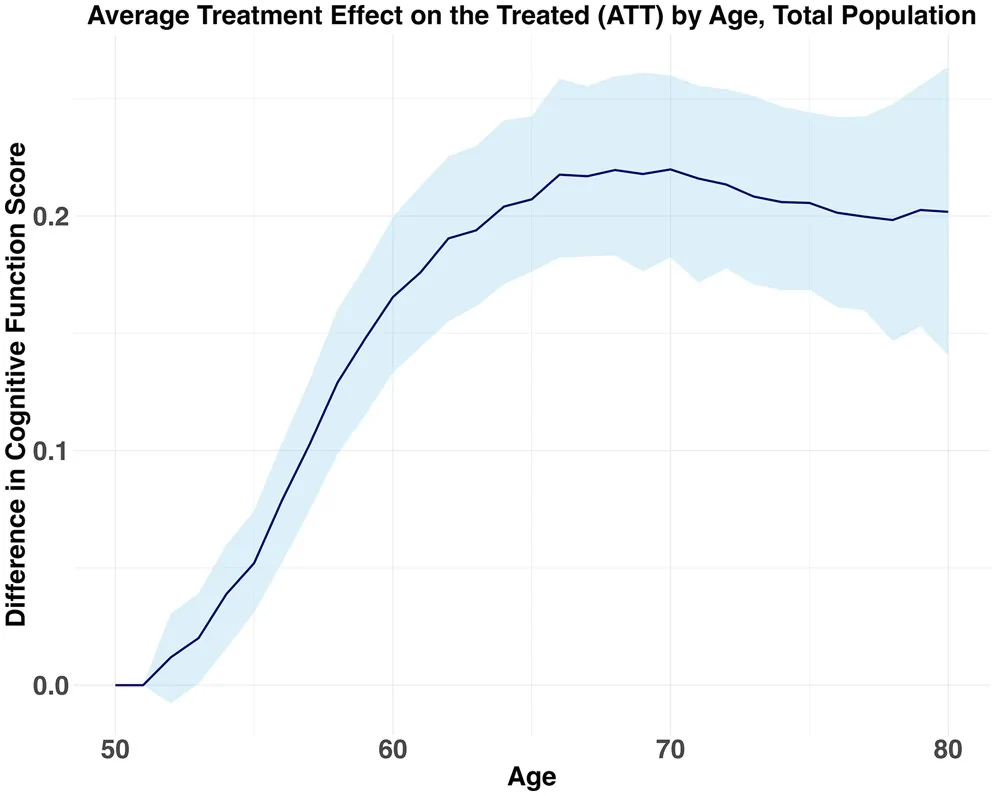
Difference in the average cognitive function score between the natural-course and hypothetical intervention scenarios among those treated, with 95% CIs.

### Moderation by gender

Figure 3 shows that TE estimates are higher for women across ages. When averaged across all ages, the TE is 0.062 (95% CI: 0.044, 0.081) for men and 0.089 (95% CI: 0.069, 0.108) for women, while the corresponding ATT values are 0.166 (95% CI: 0.136, 0.198) and 0.148 (95% CI: 0.120, 0.174), respectively, suggesting that gender does not considerably moderate the effect of loneliness on cognitive function.

**Figure 3:**
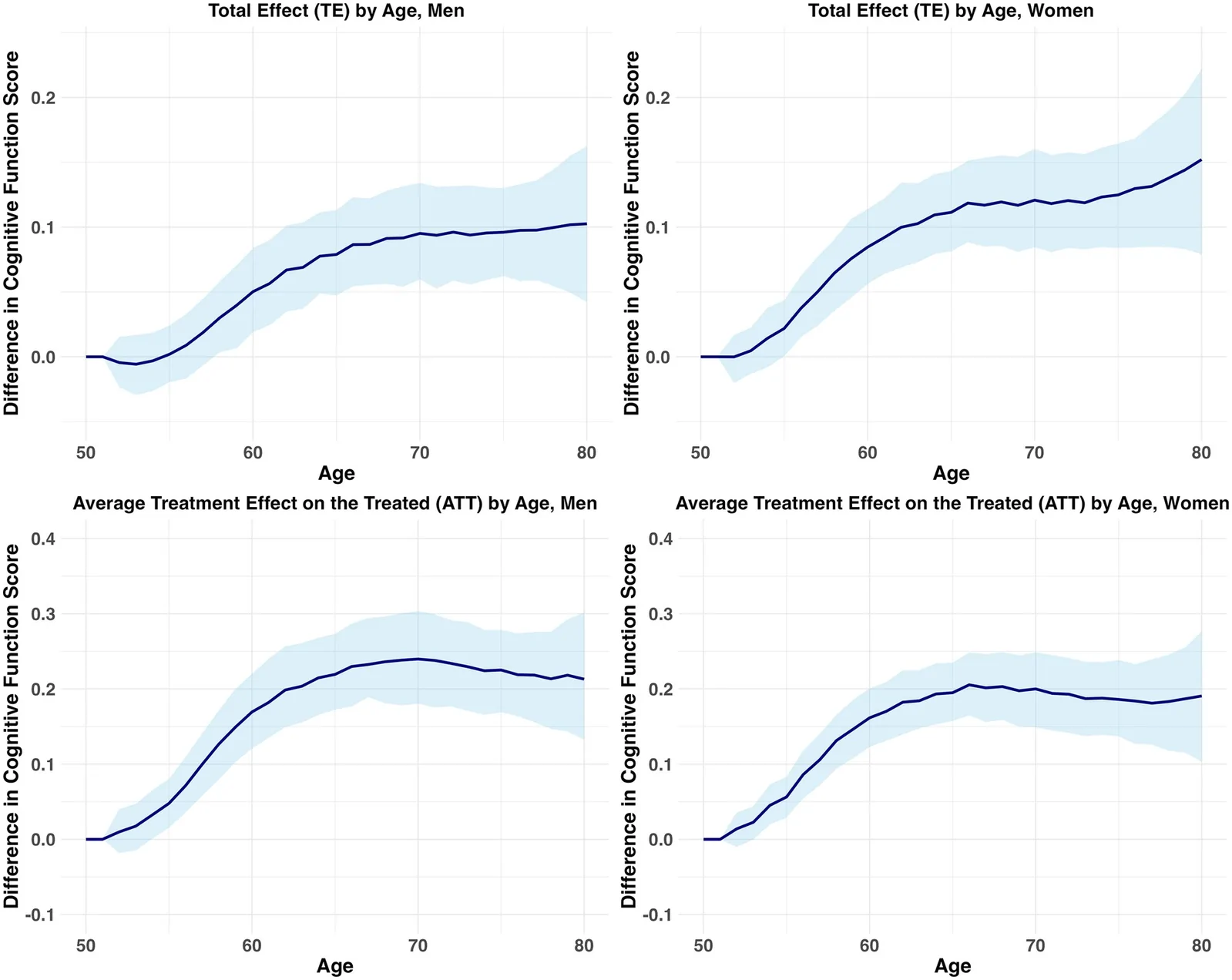
TE and ATT of preventing loneliness on cognitive function, with 95% CIs, by gender.

### Mediation via depression

Table 2 shows that depression accounts for 17.3% of the TE of preventing loneliness. The proportion mediated is greater for men (19.4%) than for women (16.9%), although most of the effect operates through other pathways.

**Table 2:** Decomposition of TE into direct effect and IE through depression, with 95% CI, by gender.

Gender	Total effect	95% CI	Direct effect	95% CI	Indirect effect	95% CI
Total	0.075	(0.063, 0.089)	0.062	(0.048, 0.076)	0.013	(0.009, 0.017)
Men	0.062	(0.044, 0.081)	0.050	(0.033, 0.070)	0.012	(0.006, 0.017)
Women	0.089	(0.069, 0.108)	0.074	(0.053, 0.093)	0.015	(0.010, 0.021)

## Discussion

The interplay of life-course risk factors shaping later-life cognitive function poses challenges, such as longitudinal interdependence, reverse causation, and intermediate confounding [16], making it difficult to isolate the effect of loneliness when using traditional regression-based methods. We have applied the parametric g-formula, which addresses these biases [17, 20], to estimate the effect of a hypothetical intervention preventing loneliness on cognitive function and to examine mediation through depression. Another key strength of this study is the use of one of the largest longitudinal samples available to examine the relationship, with the greatest number of waves spanning 22 years.

We acknowledge that several alternative causal inference approaches, including propensity-score methods, marginal structural models, and doubly robust estimators such as targeted maximum likelihood estimation (TMLE), are widely used in epidemiological research [29]. Doubly robust estimators offer an important advantage, namely consistency if either the exposure or the outcome model is correctly specified, which the parametric g-formula does not share. We nonetheless chose the parametric g-formula for reasons tied to the aims and structure of this study. First, our analysis is theory-driven rather than data-driven: we specify a causal DAG and parameterize the conditional models to follow substantive knowledge about the processes involved, whereas TMLE typically relies on flexible, data-adaptive learning of the nuisance components. Second, and most importantly, the parametric g-formula models the dynamics of the time-varying variables directly. This allows us not only to estimate the effect of the intervention, but also to examine how it propagates through the evolving process, including via other variables, which is central to our mediation analysis; standard doubly robust implementations target an estimand without modeling this dynamic structure. Third, on a practical level, the parametric g-formula was the framework we could implement with full control over the modeling choices; TMLE and related doubly robust methods for longitudinal settings remain a valuable direction for future research.

Our findings suggest that preventing loneliness could protect against later-life cognitive decline, even at the population level. This population-averaged effect is particularly useful for guiding public health strategies, as it informs about the potential cognitive benefits achievable through preventing loneliness across the population. This protective effect does not mean that preventing loneliness “boosts” cognitive function—rather, it could slow the rate of cognitive decline. The accumulating population-level cognitive benefits suggest that a dynamic loneliness intervention from midlife through older age could offer progressively greater protection against cognitive decline. Because this is a social intervention (and not pharmaceutical), the effect size of 0.07 should be contextualized accordingly.

The insulative effects of preventing loneliness on cognitive function are comparable for women and men, consistently with prior research showing no moderation in the association by gender [4]. While the population-level cognitive benefits may appear modest when averaged across the entire population, an intervention specifically targeting those who experience loneliness would have a different effect size. Therefore, to understand the individual-level impact, we also examine the ATT. The potential benefits for those directly affected are more than twice the size of the TE, underscoring the substantial impact that targeted loneliness interventions could have on cognitive health within this vulnerable subgroup. Taken together, these findings underscore the importance of addressing loneliness, not only for emotional well-being, but also as a strategy to preserve later-life cognitive function.

We have found that 17% of the protective effect of preventing loneliness on cognitive function is mediated by its impact on depression. Thus, the benefits of addressing loneliness may also extend to mental health: preventing loneliness may reduce depressive symptoms, which in turn may protect against cognitive decline. The mediating role of depression is more pronounced among men, mediating about one-fifth of the TE. This aligns with prior evidence showing that older men experience more pronounced mental health consequences associated with loneliness than do women, including higher rates of depression [14].

Our findings align with those of previous literature that varies widely in loneliness assessment (single- vs. multi-item), cognitive function measures, follow-up length, and geographic context [30]. Importantly, previous standard regression-based studies have not adequately addressed intermediate confounding or reverse causality [10]. Nevertheless, the consistency of findings across diverse methodological and contextual settings suggests a potential causal relationship. Building on this evidence, our study demonstrates that preventing loneliness could protect against later-life cognitive decline, emphasizing the importance of identifying strategies to address this serious public health concern.

Interventions enhancing social support for the bereaved or relocated, social skills, social interaction opportunities, and addressing maladaptive social cognition have shown promise in reducing loneliness [31]. Interventions are most effective when they include an educational component and target specific groups (e.g. caregivers, physically inactive people) [32]. However, Findlay [33] has reported weak evidence that existing strategies are effective for individuals who are already lonely. Future studies could use causal inference methods to compare individual and combined impacts of existing-loneliness interventions. Taken together, strategies addressing loneliness are promising public health investments, as even modest delays in cognitive impairment onset and progression could substantially reduce its global burden [7].

## Limitations

The causal interpretation of our findings relies on the assumptions of positivity, consistency, and exchangeability [34]. We offer an in-depth discussion on these assumptions in the Supplementary Materials. The HRS uses proxies for respondents who cannot complete the cognitive test. We excluded participants with proxy responses, as loneliness information is unavailable for them. As cognitively impaired individuals are more likely to require a proxy and experience loneliness, their exclusion may have led to underestimating the TE of loneliness on cognitive function at the population level.

Moreover, we relied on the single-item CES-D loneliness scale rather than the multi-item University of California, Los Angeles (UCLA) loneliness scale. However, the CES-D measure is validated and found to perform similarly to multi-item and multidimensional scales in assessing loneliness [25]. Moreover, research shows that the association between loneliness and cognitive impairment is of similar magnitude whether measured by using the CES-D or the UCLA scale [4]. Newmyer and colleagues [25] suggested that loneliness measures should be treated like self-reported health measures—useful but imperfect representation of an important concept.

## Conclusion

This study shows that preventing loneliness could provide protection against cognitive decline. The protective effects are partly mediated through reductions in depression, with this pathway appearing more relevant in men than in women. In the absence of curative treatments for cognitive impairment, social interventions against loneliness represent a promising public health strategy with additional benefits for mental health.

## Ethics approval

Written informed consent was provided by the HRS respondents. The University of Michigan Health Sciences and Behavioral Sciences Institutional Review Board approved the protocols. The data have no direct identifiers, which ensures the anonymity of participants.

## Supplementary Material

dyag212_Supplementary_Data

## Data Availability

The data used for this study are public available at https://www.rand.org/health/surveys/hrs.html. The authors make available a reproducibility package containing the analysis code. The reproducibility package is publicly available at https://osf.io/ev2ux/overview? view_only=521c4ce90dab4604a262d8c578368ed2. All analyses were implemented in R (version 4.5.2) by using the following packages: data.table, tidyverse, nnet, here, splines, fastDummies, doParallel, foreach, ggplot2, and doRNG.
