# Supplementary material for: Loneliness and cognitive function in later life: a population-based counterfactual analysis
Source: Int J Epidemiol. 2026 Oct 6;55(5):dyag212. doi: 10.1093/ije/dyag212 (PMC13641825; doi:10.1093/ije/dyag212)
Supplement: Supplementary file 1 [file dyag212_supplementary_data.docx]

# **Supplementary Materials**

Loneliness and cognitive function in later life: a population-based counterfactual analysis

**Table of Contents**

Directed Acyclic Graph (DAG)

Complementary DAG

Rationale for the First-Order Markov Dependence Structure Over Time

Step By Step Implementation of the Parametric G-Formula

Mediation Analysis: Direct and Indirect Effect

Model Assumptions

Operationalization of the Exposure Variable (Loneliness)

# **Directed Acyclic Graph (DAG)**

C_a=50_	C_a=52_	C_a=54_	... C_a=80_

L_a=50_	L_a=52_	L_a=54_	... L_a=80_

X_a=50_	X_a=52_	X_a=54_	... X_a=80_

**Figure S1**: The directed acyclic graph (DAG) shows the cross-lagged structure of the proposed relationships between loneliness (L), cognitive function (C), and time-varying confounders and mediators (X) across ages 50 to 80 years. Each arrow indicates a hypothesized causal relationship. Time-constant covariates are not shown for simplicity.

# **Complementary DAG**

**Figure** **S2**: Complementary directed acyclic graph (DAG) illustrating one full cycle of the longitudinal process

**Note**: $Z$ denotes time-constant covariates (gender, race/ethnicity, and education); $TV\_Confounder\_x$ denotes time-varying intermediate confounders (labor force status, partnership status, and comorbidity) at age x; $Depression\_x$ represents the mediator at age x; $Loneliness\_x$ is the primary exposure at age x; and $Cognition\_ x$ is the outcome of interest at age x.

**Alt Text**: Directed Acyclic Graph depicting the theoretically-derived cross-lagged longitudinal structure, showing cognitive function, loneliness, depression, and time-varying confounders at ages 50, 52, and 54.

The DAG (see Supplementary Figures S1 and S2) was developed based on literature describing the factors associated with loneliness, depression, and cognitive function, including gender^1–3^, race/ethnicity^4–6^, educational attainment^1–3^, labor force status^7–9^, partnership status^10–12^, and comorbidity^2,3,13^. Evidence on the relationships among loneliness, depression, and cognitive function^1–3,14–17^, and their influence on time-varying confounders^18–24^, informed the corresponding pathways in the DAG. Additional evidence on the interrelationships among labor force status, partnership status, and comorbidity informed the remaining causal pathways^25–27^.

# **Rationale for the First-Order Markov Dependence Structure Over Time**

In the Health and Retirement Study (HRS), which collects data biennially, a single lag already conditions on the individual’s state two years earlier, so that first-order transitions capture a substantial portion of the relevant history for the slowly-changing processes we model, such as partnership, employment, and cognition. Higher-order lags would condition on states four or more years in the past, which for persistent processes of this kind add limited predictive information once the most recent state is accounted for. First-order lags are, moreover, common in applied uses of the parametric g-formula for social exposures: Bijlsma et al. (2017)^9^ conditioned time-varying variables only on their values at the immediately preceding wave, with the aim to limit assumptions about causal ordering within a period. In studies where the lags depth has been varied, marginal estimates have been largely insensitive to it. For example, Taubman et al. (2009)^28^ found that estimates were essentially unchanged when using one or three lags instead of two. In our setting a direct comparison across lag depths is further constrained by sample size, since higher-order lags would be expected to induce sparse cells and positivity violations within subgroups defined by age, gender, education, and race/ethnicity.

Empirical studies examining the relationships between loneliness, depression, and cognitive function using cross-lagged models commonly adopt a first-order lag structure over time, which is consistent with a first-order Markov dependence assumption. For instance, Cacioppo et al. (2010)^29^ used a cross-lagged panel model to examine reciprocal relationships between loneliness and depression, with each variable predicted by its own prior value and that of the other variables at the previous wave. Similar first-order cross-lagged panel models were used by Yoon and Brown (2014)^30^ for depressive symptoms and cognitive function, Zhong et al. (2017)^17^, Bristle et al. (2025)^31^, and Cachón Alonso et al. (2023)^32^ for studying the relationship between loneliness and cognitive function.

# **Step By Step Implementation of the Parametric G-Formula**

To estimate the total effect (TE) of our hypothetical intervention for loneliness on cognitive function, we apply the g-formula using the following steps.

Step 1: Draw a random sample of adults (with replacement) from the data.

Step 2: Based on the randomly drawn sample at step 1 and following the DAG in Figure S1, estimate a series of multivariable models for the exposure, outcome, and time-varying confounders at time $t$ as a function of covariates history at $t-1$ (arrows from the time varying variables at $t-1$ to $t$ in DAG)

**A list of all parametric models used**

1) Cognitive function is a continuous variable ranging from 0 to 27 and was modelled using a linear regression model.
2) Loneliness is a binary variable with two categories: lonely vs. not lonely, and was modelled using a logistic regression model.
3) Labor force status is a categorical variable with three categories: employed (full-/part-time work), retired (retired/partly retired), and inactive (unemployed/disabled/not in labor force), and was modelled using a multinomial logistic regression model.
4) Partnership status is a binary variable with two categories: partnered (partnered/married) vs. unpartnered (separated/divorced/spouse absent/widowed/never married), and was modelled using a logistic regression model.
5) Depression is a binary variable with two categories: 0–1 vs. ≥2 Center for Epidemiological Studies-Depression (CES-D) symptoms, excluding “loneliness”, and was modelled using a logistic regression model.
6) Comorbidity is a binary variable with two categories: none vs. one or more self-reported diagnoses of diabetes, stroke, hypertension, and/or heart condition. Since transitioning back to a non-comorbid state is assumed to be impossible (absorbing state), we model the probability of *remaining* comorbidity-free using a logistic regression applied to the subset of individuals who are currently without comorbidity. The probability of transitioning into a comorbid state is then obtained as the complementary probability.

**The model expressions, functional forms, distributional assumptions, covariates, and interaction terms included in each model**

Let $Y_{g(t)}$ denote an outcome variable measured at time $t$, where g ∊ G indexes a set of variables that contains cognitive function and the following binary outcomes: lonely; partnered/unpartnered; 0-1 depressive symptoms/2+ depressive symptoms; and no morbidity/one or more morbidities. In the set G, there is also labor force status, a categorical variable with three states: employed (working full-time/part-time), retired (retired/partly retired), and inactive (unemployed/disabled/not in labor force). The set $M_{h(t-1)}$ includes the mediator variables measured at time $t-1$, with h ∊ H representing the variables included in G.

Time fixed variables are denoted as *B*_c_ where c ∊ C represents a set of variables that includes gender, race/ethnicity, and educational attainment.

Age, *A*_t,_ is measured at time *t*, modeled using a natural cubic spline with three degrees of freedom.

Finally, *L*_j(t-1)_ denotes a set of interaction terms at time *t−1*, where j ∊ J indexes the elements of set J, which includes: the interactions of cognitive function and loneliness with the age splines; the interaction between cognitive function and loneliness; the interactions of both cognitive function and loneliness with depressive symptoms; and the interaction between education and race/ethnicity. In addition, all explanatory variables and the previously defined interaction terms are further interacted with gender, which is equivalent to stratifying the model by gender. All the interactions were intended to assess potential differing patterns across the dimensions of interest.

For cognitive function, *Y*_g(t)_, a continuous outcome, we assume a normal distribution with an error term

*ε ~ N(0, σ*^2^*)* and model it as:

E[Y_g(t)_] = η + αA_t_ + Σ_c∈C_ β_c_ B_c_ + Σ_h∈H_ μ_h_ M_h(t−1)_ + Σ_j∈J_ λ_j_ L_j(t−1)_

For each binary time-varying variable, *Y*_g(t)_, including loneliness, partnership status, depressive symptoms, and comorbidities, we use a logistic regression:

logit(E[Y_g(t)_]) = η + αA_t_ + Σ_c∈C_ β_c_ B_c_ + Σ_h∈H_ μ_h_ M_h(t−1)_ + Σ_j∈J_ λ_j_ L_j(t−1)_

In these expressions, *α, β, μ*, and *λ* are the coefficients corresponding to the age spline, baseline covariates, mediating time-varying covariates, and interaction terms, respectively.

Labor force status is a categorical outcome variable with three states (employed, inactive, retired); therefore, we use a multinomial logit model to estimate the distribution of the underlying categories. Taking employed as the reference category, for each non-reference category *k* ∈ {inactive, retired}, we model:

log [ P(Y_g(t)_ = k) / P(Y_g(t)_ = employed) ] = η_k_ + α_k_A_t_ + Σ_c∈C_ β_ck_ B_c_ + Σ_h∈H_ μ_hk_ M_h(t−1)_ + Σ_j∈J_ λ_jk_ L_j(t−1)_

Each category-specific equation is estimated jointly as part of the multinomial model, and, in the g-formula simulation step, all category-specific models are simulated simultaneously.

Step 3: Define the counterfactual scenario: What would the cognitive outcome be under a counterfactual scenario where loneliness is prevented?

Step 4: From the sample in step 1, we take observations at the first time step and use the estimated models from step 2 to simulate observations for the second time step of follow-up. These simulated observations for the second time step are then used to simulate observations for the third time step, and so on until the end of follow-up^33^.

Specifically, for each synthetic individual $i$ at simulation time $t$, the values of all time-varying variables (e.g., loneliness, partnership status, employment status) are generated by simulating from the corresponding probability distributions with parameters obtained through the fitted regression models as functions of that individual’s covariate history at time $t-1$. This includes both time-constant covariates (e.g., gender, race/ethnicity) and the lagged simulated values of all time-varying covariates (e.g., lagged loneliness, partnership, employment, and cognitive function).

For binomial variables, simulated values are drawn stochastically from a binomial distribution with the predicted mean based on the corresponding multivariable model. For multinomial variables this is done the same way using a multinomial distribution. For the continuous variable (cognitive function), this is done using a Gaussian distribution (variance based on the residual variance of the prediction model)^34^.

The parametric g-formula, like other parametric methods, assumes correct model specification. In complex data structures with several time-varying variables, correctly specifying the parametric model for all those variables may be challenging^35^. Nevertheless, reporting the performance of the natural course is the most common approach to demonstrating the validity and non-misspecification of parametric models in epidemiological studies applying the parametric g-formula^35^. Following recent recommendations by Lu et al. (2025)^35^, as a model diagnostic, we compare the simulated natural course scenario with the empirical scenario for all time-varying variables at all time points in Figure S3. Although this step alone is not sufficient to validate the g-computation method, it serves as a necessary diagnostic for assessing whether our model assumptions are reasonable and is also considered good practice^36^. A reasonable agreement between the simulated natural course scenario and empirical scenario suggests that the parametric models reflect the true underlying causal mechanisms*.*

**Figure S3**. Age-specific patterns of time-varying variables- natural course (simulated) vs. observed scenario for the total population

**Alt text**: A line graph with multiple panels for cognitive function and multiple categories of the time-varying variables under study, with each panel comparing the natural course scenario with the observed/empirical scenario by age

Overall, the natural course simulations closely approximate the observed trajectories of the time-varying variables and, importantly, capture their age-related patterns. Differences between the natural course and empirical scenarios generally remain well below one-third of the y-axis range, a threshold indicative of considerable deviation^35^. Importantly, when assessing the performance of the natural course, as Lu et al. (2025)^35^ note, greater attention is placed on model fit at the left-hand side of the graphs (younger ages). Deviations toward the right-hand side, common in empirical research, receive less weight due to the reduced population size at older ages^35^. The overall close agreement between the simulated and empirical trajectories suggests that the parametric models adequately capture the underlying relationships among the time-varying variables.

Step 5*:* Save the simulated outcomes from step 4 for cognitive function and other time-varying covariates for all the follow up waves (this represents the ‘natural course’ scenario). The natural course is not exactly same as the empirical data, but should approximate it. It is a simulation of the empirical data with uncertainty (step 1 generates the uncertainty).

Step 6*:* Perform step 4 again. This time set all the “lonely” observations to “not lonely” at the first follow up wave. In the subsequent simulations, whenever “loneliness” is predicted, set it to “no loneliness.”

Step 7: Save the simulated outcomes from step 6 for cognitive function and the other time-varying covariates for all the follow up waves (this represents the counterfactual scenario).

The simulation steps in steps 4–6 are necessary because the parametric g-formula requires integrating over the joint distribution of all time-varying covariates and their past history at each time point, this high-dimensional integral cannot be computed analytically or directly summed once the number of time points and covariates grows; we therefore rely on Monte Carlo simulation, drawing repeatedly from the fitted parametric models at each time point, to approximate this integral and obtain the corresponding estimates of the counterfactual outcome distributions under each intervention of interest^37^.

Step 8: Monte Carlo error reduction: Since the simulated values are drawn from the probability distributions, we can get different values across multiple draws. This will change the effect estimates across draws. This is known as Monte Carlo error. To reduce this error, within a bootstrap iteration, we perform steps 4 to 7, 50 times, drawing a new set of time varying variable values each time. We then take the averages over them to reduce the error and produce stable estimates for natural course and counterfactual scenarios.

Step 9: Calculate the difference in average cognitive scores between the counterfactual scenario and the natural course scenario, which gives the TE of preventing loneliness on cognitive function. Save the estimate. This TE estimate is the population averaged effect if we are examining the effect in the entire population and average treatment effect on the treated (ATT) if we are examining the effect only among adults who report feeling lonely.

Step 10: Bootstrap: To produce bootstrap confidence intervals, perform steps 1 to 9, 500 times. The distribution of the TE estimates is used to obtain the mean effect. The 2.5 and 97.5% quantiles are used to obtain the 95% confidence intervals around the TE estimate.

# **Mediation Analysis: Direct and Indirect Effect**

To examine to what extent preventing loneliness influences cognitive function via depression, we perform mediation analyses. The mediation framework we use is flexible which can incorporate mediator of any type^38,39^.

First, to obtain the TE, the natural course scenario—which approximates the empirical data—is compared with the intervention scenario, in which loneliness is prevented.

Using counterfactual notation^38,39^, the natural course scenario (scenario 1) can be expressed as:

$$E[Y_{L}] = E[Y_{L,D_{L}}]$$

Where $E$ denotes the mathematical expectation, $Y$ represents the outcome variable (cognitive function), $L$ indicates loneliness status, and $D$ represents depression score.

This represents the average outcome *Y* when *L* is kept at its observed value, and *D* takes the value it has when *L* is as observed.

Using counterfactual notation, the intervention scenario (scenario 2) can be expressed as:

$$E[Y_{L^{*}}] = E[Y_{L^{*},D_{L^{*}}}]$$

Here the star (*) indicates an intervention on $L$. Since $D$ has $L^{*}$ in its subscript, this represents the value $D$ would take when there is an intervention on $L$.

If we subtract the average cognitive function score in the natural course scenario from the average cognitive function score in the intervention scenario, we obtain the TE.

Using counterfactual notation, the TE can be expressed as:

$$E\left[ Y_{L^{*},D_{L^{*}}} \right]- E\left[ Y_{L,D_{L}} \right]$$

For the mediation analysis, we simulate another scenario. In that scenario, the intervention (preventing loneliness) is performed, but the mediating variable—depressive symptoms—is kept at the natural course levels^38,39^. In this setup, the intervention will not influence the mediator, hence the intervention will not operate via the mediating variable onto cognitive function (outcome variable). The intervention will only operate on cognitive function directly and via mediating variables that are not kept at their natural course levels.

In this third scenario (scenario 3), the depression score is kept at the natural course levels. Using counterfactual notation, this can be expressed as:

$$E[Y_{L^{*},D_{L}}]$$

Using counterfactual notation, we calculate the total direct effect (TDE) of our intervention (not through depression score) as:

$$E\left[ Y_{L^{*},D_{L}} \right]-E[Y_{L,D_{L}}]$$

The pure indirect effect (PIE) of our intervention operating through the depression score is defined as the difference between the TE and TDE that does not operate through the depression score^38,39^. It is calculated as:

$$\left\{ \left. E\left[ Y_{L^{*},D_{L^{*}}} \right]- E\left[ Y_{L,D_{L}} \right] \right\} \right.- \left\{ \left. E\left[ Y_{L^{*},D_{L}} \right]-E[Y_{L,D_{L}}] \right\} \right.$$

$E\left[ Y_{L^{*},D_{L^{*}}} \right]$ $-$ $E\left[ Y_{L^{*},D_{L}} \right]$

This PIE should be interpreted as to what extent preventing loneliness affects cognitive function via depression only (i.e., due to loneliness affecting depression and subsequently, depression affecting cognitive function).

# **Model Assumptions**

As with all empirical studies, our findings are grounded in a specific set of underlying assumptions, including appropriate temporal ordering and the key causal assumptions of positivity, consistency, and exchangeability^40^. Temporal ordering is addressed through the cross-lagged framework, which establishes a clear temporal sequence and helps reduce the chance of bias from reverse causality.

Positivity, together with exchangeability and consistency, is required to identify causal estimands. It states that within every stratum of the observed confounders Z, each exposure level occurs with positive probability: whenever f(z) > 0, then Pr(A = a | Z = z) > 0 for all a. Structural (deterministic) violations occur when a rule or constraint makes an exposure impossible for a subgroup. For instance, in a study investigating loneliness among individuals aged 50 to 80, a structural violation would occur if the exposure of interest is enrollment in state-funded senior social centers that strictly require a minimum age of 65. Consequently, for the subgroup aged 50–64, the probability of exposure is deterministically zero, as it would be impossible to contrast outcomes between exposed and unexposed individuals within that age stratum. Random (stochastic) violations arise from finite-sample sparsity, as when no treated individuals happen to fall in a stratum defined by the covariate. For example, there are no exposed individuals within a stratum defined by age 80. The empty cell(s) is a consequence of small sample size, and the common solution is to collapse adjacent strata, use parametric modeling to extrapolate across strata, and when possible obtain a larger sample size.

**The positivity assumption** requires that each individual in our study must have a non-zero probability of receiving the intervention, meaning that there are no covariate-defined strata in which individuals are either exclusively treated or exclusively untreated, ensuring adequate overlap in treatment assignment across strata. In this study, we consider a hypothetical intervention of preventing loneliness, assuming that it is feasible for individuals to transition from being lonely to non-lonely. If loneliness cannot be prevented for some individuals, the intervention is not well defined for them, and the population-averaged effect should be read as applying to those for whom prevention is feasible rather than to the whole population. This concern is substantially attenuated for the ATT, which is estimated among individuals who are lonely at least once during follow-up. The ATT requires only one-sided overlap: it needs comparable non-lonely counterfactuals for these individuals, but is unaffected by strata in which loneliness never occurs, and is therefore less exposed to positivity violations than the population-averaged effect.

**To assess the plausibility of the positivity assumption**, we examined the distribution of propensity scores for loneliness. No predicted probabilities were equal to 0 or 1, and there was substantial common support, with predicted probabilities ranging from 0.014 to 0.849 among lonely individuals and from 0.012 to 0.857 among non-lonely individuals.

However, since the parametric g-formula does not signal positivity violations through unstable behaviour, but instead absorbs them into model-based extrapolation, such violations must be addressed at the level of the specification rather than diagnosed from the estimator’s output. **As a robustness check**, we therefore re-estimated the g-formula under a coarser covariate specification, aggregating categories to reduce the number of confounder strata and hence the occurrence of sparse or empty cells that give rise to random non-positivity. Specifically, we collapsed partnership status into partnered (partnered or married) versus unpartnered (never married, separated/divorced/spouse absent, or widowed); grouped labour-force status into inactive (not in the labour force, unemployed, or disabled), retired (retired/partly retired), and employed (full-time/part-time work); and reduced comorbidity and depression to binary indicators of presence versus absence. We believe this coarsening is defensible because any sparse cells in comorbidity would reflect finite-sample sparseness rather than a structural zero, and because depression enters the simulation as a mediator drawn from its distribution as observed in the natural course, so that the granularity of its categories does not affect the estimated marginal effect.

Results were essentially unchanged, both qualitatively and quantitatively. Since coarsening covariates trades a potential reduction in non-positivity bias against a potential increase in residual confounding, the stability of the estimates indicates that this trade-off is not consequential here. We, therefore, in the revised manuscript, report results under the coarser specification, which is less exposed to random positivity violations.

**The consistency assumption** requires that the hypothetical intervention is clearly and consistently defined so that it accurately reflects the intervention’s effect. In our study, loneliness is operationalized using the single item of the CES-D loneliness measure and the intervention corresponds to setting lonely individuals to the non-lonely state. The CES-D measure, a widely used validated loneliness measure, has been shown to tap into the same underlying construct of loneliness similarly by gender and age in diverse societies, including the US^41^, indicating consistent exposure measurement across all study participants.

We acknowledge that loneliness may arise from distinct emotional and social dimensions, which can be understood as different versions of the exposure. In such a case, it would be ideal to analyze them separately to avoid violations of the consistency assumption. However, the HRS does not allow such a distinction. As in all observational studies, consistency is therefore not fully guaranteed^42^. Under multiple exposure versions, however, the quantity we estimate has a clear interpretation: it is the effect of loneliness averaged over the distribution of its emotional and social components as they occur in the study population. Because any intervention in the real world will act on loneliness as it is actually distributed rather than on any single dimension, the average effect is policy relevant^42^.

Although there are various reasons for individuals’ loneliness, we aim to achieve comparability between lonely and non-lonely individuals to support the exchangeability assumption required for causal interpretation of our findings. **The exchangeability assumption** requires adjustment for all relevant confounders so that lonely and non-lonely individuals are comparable conditional on measured covariates. Based on prior literature, we adjust for an extensive set of confounders covering social, demographic, socioeconomic, psychosocial, and health-related domains. **We further tested the robustness of our estimates to the modelling choices**. We added covariates such as birth cohort; we varied the functional form for age, including a cubic polynomial specification; we truncated the analysis at 2018 to avoid distortions related to the COVID-19 pandemic; and we modelled comorbidity and depression using a Firth penalized-likelihood approach as an alternative to the main specification. Across all of these variations the results were stable in both direction and magnitude. **Despite the computational complexity of the approach, we therefore tested the specification, functional forms, and variable inclusion extensively, and the conclusions were unchanged throughout**. Nevertheless, as in all observational research, residual confounding due to unobserved or imprecisely measured variables may remain. Consequently, we cannot fully rule out bias in our causal estimates.

Despite these limitations, which are common across other analytical approaches, the parametric g-formula provides a flexible and powerful framework for addressing key challenges in epidemiological analyses. By applying this framework to a hypothetical intervention against loneliness, we contribute to the limited literature using the g-formula for social exposures^35^. Furthermore, by making our code and software publicly available, we aim to facilitate its wider application in social epidemiology.

# **Operationalization of the Exposure Variable (Loneliness)**

Loneliness can be assessed using different self-reported measures, each with strengths and limitations. The HRS includes two such measures: a single-item loneliness indicator embedded within the CES-D scale and the University of California, Los Angeles (UCLA) Loneliness Scale. Both are widely used unidimensional measures of loneliness^41^. In the CES-D measure, respondents are asked whether they felt lonely during the past week (yes/no) much of the time. Compared with the UCLA scale, a limitation of the CES-D item is that it explicitly uses the term “lonely,” which may lead to underreporting due to stigma associated with loneliness^43^. Nevertheless, evidence from over 30 countries indicates high consistency across available loneliness measures, despite differences in reference periods and wordings of questions, and supports that loneliness can be measured in a robust, valid, and reliable manner using single-item measures, including the CES-D measure^41,44^.

Importantly, in the HRS, the CES-D loneliness item has been available since study inception, whereas the UCLA scale has only been administered since 2006/2008, administered to 50% of the sample at alternate waves, yielding data once every four years per respondent. This substantially reduces sample size. Since the parametric g-formula is sensitive to small sample sizes^45^, using the UCLA scale would likely result in unstable estimates, sparse cell problems for covariate strata, and wider confidence intervals. Therefore, we use the binary CES-D loneliness measure, which has comparable capacity to capture loneliness as the UCLA Scale^41,44^ and is biennially available for the full sample, ensuring a long follow-up period from 1996 to 2018 with sufficient statistical power and model stability.

A substantial body of literature using the CES-D loneliness measure among adults aged 50+ has documented considerable associations with cognitive function, cognitive decline, and cognitive impairment, including in analyses based on the HRS^1,46–49^. Importantly, the ‘past week’ reference period does not reflect the duration of exposure (transient vs. persistent loneliness) and should be interpreted as an indicator of the respondent’s current loneliness state at each wave. Empirical evidence suggests that both transient and chronic loneliness are associated with cognitive function and decline^50,51^.

Furthermore, Salinas et al. (2022)^52^, using data from the Framingham Study, showed that the CES-D past-week loneliness was associated with early markers of vulnerability to neurocognitive decline in adults aged 40–79, including lower total cerebral volume and greater white matter hyperintensities. These findings provide biological plausibility for pathways linking loneliness to neurocognitive decline that precedes clinically manifest cognitive impairment.

The single-item CES-D measure is also advantageous in large-scale surveys due to its low response burden and has been suggested to be particularly suitable for older population, including individuals with poorer cognition, compared with longer instruments such as the UCLA scale^53,54^.

# **References**

**1.	Donovan NJ, Wu Q, Rentz DM, Sperling RA, Marshall GA, Glymour MM. Loneliness, depression and cognitive function in older US adults. International journal of geriatric psychiatry. 2017 May;32(5):564-73.**

**2.	Livingston G, Huntley J, Sommerlad A, Ames D, Ballard C, Banerjee S, Brayne C, Burns A, Cohen-Mansfield J, Cooper C, Costafreda SG. Dementia prevention, intervention, and care: 2020 report of the Lancet Commission. The lancet. 2020 Aug 8;396(10248):413-46.**

**3.	Zivin K, Llewellyn DJ, Lang IA, Vijan S, Kabeto MU, Miller EM, Langa KM. Depression among older adults in the United States and England. The American Journal of Geriatric Psychiatry. 2010 Nov 1;18(11):1036-44.**

**4.	Hawkley LC, Hughes ME, Waite LJ, Masi CM, Thisted RA, Cacioppo JT. From social structural factors to perceptions of relationship quality and loneliness: the Chicago health, aging, and social relations study. The Journals of Gerontology Series B: Psychological Sciences and Social Sciences. 2008 Nov 1;63(6):S375-84.**

**5.	Reuser M, Willekens FJ, Bonneux L. Higher education delays and shortens cognitive impairment. A multistate life table analysis of the US Health and Retirement Study. European journal of epidemiology. 2011 May;26(5):395-403.**

**6.	Bailey RK, Mokonogho J, Kumar A. Racial and ethnic differences in depression: current perspectives. Neuropsychiatric disease and treatment. 2019 Feb 22:603-9.**

**7.	Morrish N, Medina-Lara A. Does unemployment lead to greater levels of loneliness? A systematic review. Social science & medicine. 2021 Oct 1;287:114339.**

**8.	Hale JM, Bijlsma MJ, Lorenti A. Does postponing retirement affect cognitive function? A counterfactual experiment to disentangle life course risk factors. SSM-Population Health. 2021 Sep 1;15:100855.**

**9.	Bijlsma MJ, Tarkiainen L, Myrskylä M, Martikainen P. Unemployment and subsequent depression: A mediation analysis using the parametric G-formula. Social Science & Medicine. 2017 Dec 1;194:142-50.**

**10.	Hawkley LC, Wroblewski K, Kaiser T, Luhmann M, Schumm LP. Are US older adults getting lonelier? Age, period, and cohort differences. Psychology and aging. 2019 Dec;34(8):1144.**

**11.	Håkansson K, Rovio S, Helkala EL, Vilska AR, Winblad B, Soininen H, Nissinen A, Mohammed AH, Kivipelto M. Association between mid-life marital status and cognitive function in later life: population based cohort study. Bmj. 2009 Jul 2;339.**

**12.	Hald GM, Ciprić A, Sander S, Strizzi JM. Anxiety, depression and associated factors among recently divorced individuals. Journal of Mental Health. 2022 Jul 4;31(4):462-70.**

**13.	Lim MH, Manera KE, Owen KB, Phongsavan P, Smith BJ. The prevalence of chronic and episodic loneliness and social isolation from a longitudinal survey. Scientific Reports. 2023 Aug 1;13(1):12453.**

**14.	Guarnera J, Yuen E, Macpherson H. The impact of loneliness and social isolation on cognitive aging: a narrative review. Journal of Alzheimer's disease reports. 2023 Jun 29;7(1):699-714.**

**15.	Harrington KD, Vasan S, Kang JE, Sliwinski MJ, Lim MH. Loneliness and cognitive function in older adults without dementia: A systematic review and meta-analysis. Journal of Alzheimer’s Disease. 2023 Feb 14;91(4):1243-59.**

**16.	Luo M. Social isolation, loneliness, and depressive symptoms: a twelve-year population study of temporal dynamics. The Journals of Gerontology: Series B. 2023 Feb 1;78(2):280-90.**

**17.	Zhong BL, Chen SL, Tu X, Conwell Y. Loneliness and cognitive function in older adults: Findings from the Chinese longitudinal healthy longevity survey. Journals of Gerontology Series B: Psychological Sciences and Social Sciences. 2017 Jan 1;72(1):120-8.**

**18.	Morrish N, Mujica-Mota R, Medina-Lara A. Understanding the effect of loneliness on unemployment: propensity score matching. BMC Public Health. 2022 Apr 28;22(1):740.**

**19.	Wang L, Pan H, Cai Z, Li X, Luo L. Association of social isolation, loneliness and risk of cardiovascular diseases: Meta-analysis of cohort studies. BMC Public Health. 2025 Sep 24;25(1):3082.**

**20.	Ciliacus R, Hijdra RW, Robroek SJ, Leist AK, Burdorf A, Schuring M. Memory function and early exit from paid employment through different pathways among ageing European workers. Scandinavian journal of work, environment & health. 2025 Feb 28;51(2):89.**

**21.	Zheng W, Schimmele CM, Penning MJ, Zheng C, Noh S. Effect of marital status on duration of treatment for mental illness.**

**22.	Leng X, Espeland MA, Manson JE, Stefanick ML, Gower EW, Hayden KM, Limacher MC, Vaughan L, Robinson J, Wallace R, Wassertheil-Smoller S. Cognitive function and changes in cognitive function as predictors of incident cardiovascular disease: the Women’s Health Initiative Memory Study. The Journals of Gerontology: Series A. 2018 May 9;73(6):779-85.**

**23.	Wu J, Tian Z, Qi Z, Liu X, Yu P. Cardiovascular disease and depression: a bidirectional relationship and its clinical implications. Frontiers in Psychiatry. 2026;16:1675680.**

**24.	Whooley MA, Kiefe CI, Chesney MA, Markovitz JH, Matthews K, Hulley SB. Depressive symptoms, unemployment, and loss of income: The CARDIA Study. Archives of Internal Medicine. 2002 Dec 9;162(22):2614-20.**

**25.	Zhang Z, Gong Q, Gilleskie D, Moulton JG, Sylvia SY. The impact of multimorbidity on labor force participation among the middle-aged and older working population in the United States. The Journals of Gerontology, Series B: Psychological Sciences and Social Sciences. 2024 Oct;79(10):gbae131.**

**26.	Ramezankhani A, Azizi F, Hadaegh F. Associations of marital status with diabetes, hypertension, cardiovascular disease and all-cause mortality: a long term follow-up study. PloS one. 2019 Apr 22;14(4):e0215593.**

**27.	Jalovaara M. The joint effects of marriage partners’ socioeconomic positions on the risk of divorce. Demography. 2003 Feb;40(1):67-81.**

**28.	Taubman SL, Robins JM, Mittleman MA, Hernán MA. Intervening on risk factors for coronary heart disease: an application of the parametric g-formula. International journal of epidemiology. 2009 Dec 1;38(6):1599-611.**

**29.	Cacioppo JT, Hawkley LC, Thisted RA. Perceived social isolation makes me sad: 5-year cross-lagged analyses of loneliness and depressive symptomatology in the Chicago Health, Aging, and Social Relations Study. Psychology and aging. 2010 Jun;25(2):453.**

**30.	Yoon JY, Brown RL. Causal inference in cross-lagged panel analysis: A reciprocal causal relationship between cognitive function and depressive symptoms. Research in Gerontological Nursing. 2014 Jul 1;7(4):152-8.**

**31.	Bristle J, Wagner M, Hannemann TV, Cohn-Schwartz E. Loneliness and cognitive decline in ageing Europe: Does country context matter for memory performance?. Journal of Public Health. 2025 May 30:1-3.**

**32.	Cachón-Alonso L, Hakulinen C, Jokela M, Komulainen K, Elovainio M. Loneliness and cognitive function in older adults: Longitudinal analysis in 15 countries. Psychology and aging. 2023 Dec;38(8):778.**

**33.	Keil AP, Edwards JK, Richardson DB, Naimi AI, Cole SR. The parametric g-formula for time-to-event data: intuition and a worked example. Epidemiology. 2014 Nov 1;25(6):889-97.**

**34.	Robins J. A new approach to causal inference in mortality studies with a sustained exposure period—application to control of the healthy worker survivor effect. Mathematical modelling. 1986 Jan 1;7(9-12):1393-512.**

**35.	Lu W, Picciotto S, Costello S, Colbeth H, Eisen E. Evaluating natural course performance in parametric g-formula: review of current practice and illustration based on the United Autoworkers–General Motors cohort. American Journal of Epidemiology. 2025 Aug;194(8):2249-60.**

**36.	Rudolph JE, Cartus A, Bodnar LM, Schisterman EF, Naimi AI. The role of the natural course in causal analysis. American journal of epidemiology. 2022 Feb;191(2):341-8.**

**37.	Westreich D, Cole SR, Young JG, Palella F, Tien PC, Kingsley L, Gange SJ, Hernán MA. The parametric g‐formula to estimate the effect of highly active antiretroviral therapy on incident AIDS or death. Statistics in medicine. 2012 Aug 15;31(18):2000-9.**

**38.	Wang A, Arah OA. G-computation demonstration in causal mediation analysis. European journal of epidemiology. 2015 Oct;30(10):1119-27.**

**39.	Bijlsma MJ, Wilson B. Modelling the socio-economic determinants of fertility: a mediation analysis using the parametric g-formula. Journal of the Royal Statistical Society Series A: Statistics in Society. 2020 Feb;183(2):493-513.**

**40.	Greenland S, Robins JM. Identifiability, exchangeability and confounding revisited. Epidemiologic Perspectives & Innovations. 2009 Sep 4;6(1):4.**

**41.	Newmyer L, Verdery AM, Margolis R, Pessin L. Measuring older adult loneliness across countries. The Journals of Gerontology: Series B. 2021 Sep 1;76(7):1408-14.**

**42.	VanderWeele TJ, Hernan MA. Causal inference under multiple versions of treatment. Journal of causal inference. 2013 May 29;1(1):1-20.**

**43.	Victor C, Grenade L, Boldy D. Measuring loneliness in later life: A comparison of differing measures. Reviews in Clinical Gerontology. 2005 Feb;15(1):63-70.**

**44.	Mund M, Maes M, Drewke PM, Gutzeit A, Jaki I, Qualter P. Would the real loneliness please stand up? The validity of loneliness scores and the reliability of single-item scores. Assessment. 2023 Jun;30(4):1226-48.**

**45.	Keil AP, Daza EJ, Engel SM, Buckley JP, Edwards JK. A Bayesian approach to the g-formula. Statistical methods in medical research. 2018 Oct;27(10):3183-204.**

**46.	Luchetti M, Terracciano A, Aschwanden D, Lee JH, Stephan Y, Sutin AR. Loneliness is associated with risk of cognitive impairment in the Survey of Health, Ageing and Retirement in Europe. International journal of geriatric psychiatry. 2020 Jul;35(7):794-801.**

**47.	Sutin AR, Stephan Y, Luchetti M, Terracciano A. Loneliness and risk of dementia. The Journals of Gerontology: Series B. 2020 Aug 13;75(7):1414-22.**

**48.	Lee JJ, Park MK, Kim N, Kim L, Kim GS. Longitudinal relationship between baseline social frailty and cognitive impairment in older adults: 14-year follow-up results from the Korean longitudinal study of ageing. Journal of the American Medical Directors Association. 2024 Sep 1;25(9):105124.**

**49.	O’Luanaigh C, O’Connell H, Chin AV, Hamilton F, Coen R, Walsh C, Walsh JB, Caokley D, Cunningham C, Lawlor BA. Loneliness and cognition in older people: The Dublin Healthy Ageing study. Aging & mental health. 2012 Apr 1;16(3):347-52.**

**50.	Zhong BL, Chen SL, Conwell Y. Effects of transient versus chronic loneliness on cognitive function in older adults: Findings from the Chinese Longitudinal Healthy Longevity Survey. The American Journal of Geriatric Psychiatry. 2016 May 1;24(5):389-98.**

**51.	Martín-María N, Caballero FF, Miret M, Tyrovolas S, Haro JM, Ayuso-Mateos JL, Chatterji S. Differential impact of transient and chronic loneliness on health status. A longitudinal study. Psychology & Health. 2020 Feb 1;35(2):177-95.**

**52.	Salinas J, Beiser AS, Samra JK, O'Donnell A, DeCarli CS, Gonzales MM, Aparicio HJ, Seshadri S. Association of loneliness with 10-year dementia risk and early markers of vulnerability for neurocognitive decline. Neurology. 2022 Mar 29;98(13):e1337-48.**

**53.	Russell D, Peplau LA, Cutrona CE. The revised UCLA Loneliness Scale: concurrent and discriminant validity evidence. Journal of personality and social psychology. 1980 Sep;39(3):472.**

**54.	Fees BS, Martin P, Poon LW. A model of loneliness in older adults. The Journals of Gerontology Series B: Psychological Sciences and Social Sciences. 1999 Jul 1;54(4):P231-9.**
